# Personality Factors Underlying Suicidal Behavior Among Military Youth

**DOI:** 10.5812/ircmj.12686

**Published:** 2014-04-05

**Authors:** Abdollah Soltaninejad, Ali Fathi-Ashtiani, Khodabakhsh Ahmadi, Hediye Sadat Mirsharafoddini, Alireza Nikmorad, Motahare Pilevarzadeh

**Affiliations:** 1 Behavioral Sciences Research Center, Baqiyatallah University of Medical Sciences, Tehran, IR Iran; 2 Department of Economic, Firoozkooh Branch, Islamic Azad University, Firoozkooh, IR Iran; 3 Nursing and Midwifery Faculty, Jiroft University of Medical Sciences, Jiroft, IR Iran

**Keywords:** Personality, Military Personnel, Behavior, Suicide

## Abstract

**Background:**

Suicidal behavior is one the most significant mental health problems in the military. Militaries are closed systems that operate in particular situations. Military service is associated with certain stressful conditions. On this basis, there is likely of trauma in the military environment. Measures of suicidal behavior are pathologically complex. A range of biological, psychological, social, and institutional factors are involved in the incidence and prevalence of these behaviors.

**Objectives:**

One of the underlying factors in suicidal behavior is individuals' personality.

**Patients and Methods:**

The study population comprised of the Iranian Armed Forces. To recruit the sample of the research, 1659 soldiers were selected by multistage sampling. Data were collected using the Beck Scale for Suicide Ideation (BSSI) and NEO-Five Factor Inventory.

**Results:**

There was a significant positive correlation (r = 0.323) between neuroticism and suicide ideation; however, significant negative correlations existed between three other personality traits --extraversion [r = -0.306], agreeableness [r = -0.227], and conscientiousness [r = -0.271] and suicidal ideation. Unlike neuroticism, extraversion and conscientiousness personality factors could reduce significantly (as much 14% as are predicted) levels of suicidal ideation.

**Conclusions:**

Based on these results, neuroticism might increase suicide, but extraversion and conscientiousness personality traits are associated with a reduced risk of suicide.

## 1. Background

Suicide is one of the ten leading causes of death in the general population and the second or third leading cause of death among 15 to 34 years-old people (1). Average Global rate of suicide is 16 per 100000 people. A decade ago, it was estimated that the disease burden due to suicide was 1.8% of the total diseases. New estimations suggest the growth of the disease burden, and this figure is likely to reach 2.4% in 2020. Overall, suicides have increased about 60% during the past half century (2). The World Health Organization estimates that in 2020 approximately 1530000 people will die due to suicide, and the number of attempted suicide will be 10 to 20 times more. Deaths from suicide in both sexes and in children and adults alike have increased during the past forty-five years (3). This organization also reports that suicide contributes to the largest deliberate damage in the developed countries and in the coming decades the future burden of disease will increase (4).

 Suicidal behavior refers to a range of behaviors that are similar in the deliberate intent to kill oneself. Suicidal ideation, plan and attempt constitute three important suicidal behaviors. Suicidal ideation is one of the strongest predictors of suicide. Studies have shown that most suicides occur when people think about it (5). Suicidal behaviors are pathologically complex and a wide range of biological, psychological, social, cultural and spiritual factors are involved in it. 

Suicide in the military is an important mental health issue due to several reasons such as access to weapons, familiarity to use them, and high-stress situations. Although armed forces have appropriate mental health and physical shape and are repeatedly screened for mental diseases, the suicidal risk factors (youth, access to weapons, stress, special situations and aggressive individuals in the military) would predispose them to suicide. 

Some experts believe that the suicide rate in the army should be lower than that of the general population (6) due to the suicide screening programs and access to mental health services. But other conflicting points of view claim that, aggression and access to guns increase the risk of suicide in the military. Research evidence seems to support the latter view (7). Suicidal behavior at any extent and in any manner has devastating effects on the mental health of the individual, family, survivor, and administrators. There are considerable evidences in the mental health literature suggesting that certain personality factors are associated with increased suicidal risk (8). 

Personality factors are factors, which are determined through gene-gene and gene-environment interactions (9). Depending on the theoretical and methodological approaches, personality factors are listed as follows: extraversion, introversion, agreeableness, modernistic, enthusiasm, rewards associated, conscientious, compatibility, and neuroticism. Suicidal temperament hypothesizes that certain personality factors may make the person vulnerable to suicide. Mood, self-destructive tendency, and some characteristics such as anger, aggression and anxiety are interrelated (10). Clinical and epidemiologic studies have shown that certain personality factors such as impulsivity, negativity, introversion, avoidance, dependence, neuroticism and antisociality are associated with suicidal behavior. These personality factors may underlie some cluster B personality disorders, which are important risk factors for suicidal behavior (10). 

Studies have also proven the relationship between a number of certain personality factors and neurotransmitters such as serotonin and GABA (gamma-aminobutyric acid). In psychology literature, impairment in neurotransmitters is confirmed as one of the involved suicidal hypotheses (11). The role of serotonin is more important than other neurotransmitters (12). Some studies have shown that people with anxious personality are manipulated in less stressful situations. These people are less likely to seek help or escape when faced with a critical situation. Such people, faced with stressful situations feel desperate due to their poor judgments. In such circumstances that they feel, there is no escape, and the feeling of helplessness may fuel self-defeating behaviors carrier shall provide (13). 

Five factor model of personality suggests that personality characteristics can be categorized into five main personalities: Neuroticism, Extraversion, Openness, Agreeableness, and Conscientiousness. Empirical evidence has shown that there is a relationship between certain personality traits and suicidal thoughts. For example, neuroticism personality dimension is associated with negative emotions like depression and tendency to depression is considered a risk factor for suicide (14). Studies have also shown that suicidal ideation is associated with low levels of extraversion which may reflect a low tendency to experience positive emotions (15).

 Identification of risk factors of suicidal attempts is the most significant preventive program in militaries across the world. Few studies have examined the relationship between personality factors and suicide in non-clinical samples and most studies have been conducted on patients.

## 2. Objectives

The present study aims at investigating the role of personality factors in the prediction of suicide ideation among Iranian young soldiers.

## 3. Patients and Methods

This study is a correlation type. The study population consisted of all soldiers serving in some military organizations in Iran during 2012. High school educated, being solider and willingness to participate were inclusion criteria and having a mental illness was the exclusion criterion.

To determine the sample, 1,463 soldiers in six Provinces: Ilam; Tehran; Sistan and Baluchestan; Hormozgan; Kermanshah; and Mazandaran, were selected using multistage cluster sampling. A military facility was selected in each province. Regarding the number of soldiers of each unit, a sample was randomly selected. The following formula is used to calculate the sample size:

*n* = ((Z_ (α/2)_ + Z_β_)^2^ P(1-P)) / δ^2^

Where α: Type I error, β: Type II error and Z_p_: is quintile of standard normal distribution with probability p.

Considering α = 0.05 (Z_0.05_ = 1.63), β = .1 (Z_0.1_ = 1.28), P = .06 and precision δ = 0.022 the sample size is calculated as follows:

*n* = ((Z_(α/2)_ + Z_β_)^2^ P(1-P)) / δ^2^ = (10.49 × 0.06(0.94)) / 0.022^2^ = 0.591 / 0.0005 = 1182

Assuming 23% falling in the sample, totally we have 1182 + 272 = 1454.

Medical Ethics Committee of Behavioral Sciences Research Center of Baqiyatallah University of Medical Sciences approved the study (code number: 374, Date: May 11, 2011).

Data were analyzed using SPSS software version 17. Correlation analysis was also used to study the association between two continuous variables in this survey. Regression Analysis was used to see the effect of personality factors on suicide ideation. P-values less than .05 were considered statistically significant.

### 3.1. Beck Scale for Suicide Ideation

Beck Scale for Suicide Ideation (BSSI) is a 19-question self-reporting tool designed to detect and measure the severity of suicidal attitudes and behaviors as well as plans to commit suicide. Questions of this scale measure items such as death wish, suicidal tendencies as active and inactive, duration and frequency of suicide ideation, feelings of self-control, and preventive factors for suicide and individual’s level of readiness to attempt suicide. This questionnaire contains 19 three-option items; set according to the degree point of zero to two. Individual's overall score is calculated based on the total score varying from zero to 38. Validity of the scale, using Cronbach's alpha, has been reported to be 0.95 (16).

### 3.2. NEO Personality Inventory

NEO Five-Factor Inventory (NEO-FFI) is a self-reporting personality type questionnaire designed based on a five-factor model. The questionnaire contains 60 questions measuring five dimensions of personality factors, including neuroticism (N), extraversion (E), openness to experience (O), agreeableness (A) and conscientiousness (C). Costa and McCrae (1992) have reported that the reliability of the scale using Cronbach's alpha for the subscales is 0.68 to 0.86 (17). Garooci and his colleagues investigated validity and reliability of the Iranian version of NEO-FFI. They reported Cronbach's alpha coefficients for each of the main factors of Neuroticism, Extraversion, Openness, Agreeableness and Conscientiousness, as 0.86, 0.73,0.56,0.68 and 0.87 respectively (18).

## 4. Results

Out of 1,463 soldiers studied, 397 (27.1%) had junior high school education, 145 (9.9 %) senior high school education, 537 (36.7%) high school diploma, 246 (16.8%) associate degree and 138 (9.4%) B.A or B.S. Subjects had a mean age of 22 ± 7.2 years. Of them, 571 (39%) were under twenty, 731 (50%) were 25-21 years old and 161 (26%) were older than 26. Eighty-six-point-seven percent of the sample group were single, 12.2% were married, and 1.2% were divorced.

 According to Table 2, the correlation coefficient analysis showed a significant positive correlation between suicide ideation and neuroticism. Personality factors, including extraversion, openness, and agreeableness showed a negative significant correlation with suicide ideation. It was also revealed that openness personality factor was not correlated with suicide ideation. 

Table 3, presents the coefficient of multiple correlations between personality factors and suicide ideation, coefficient of determination (the level of variability in the dependent variable that can be explained by the regression model), adjusted coefficient of determination, and standard error estimates. According to the results presented in Table 4, the significance of two models is assured. 

Table 5 summarizes the common and standard regression analysis coefficients, standard deviation, statistic, and the significance level of the model. Considering the coefficients of the three models, positive and significant relationships between all variables are assured. Also, the test statistic can be used to prove that neuroticism has a greater impact on the prediction of suicide ideation than extraversion and conscientiousness.

**Table 1.** Demographic Data of the Sample ^a^

	Number (Frequency)
**Education**	
Junior high school	397 (27.1)
Senior high school	145 (9.9)
Diploma graduate	537 (36.7)
Associate degree	246 (16.8)
BA/BS	138 (9.4)
Total	1463 (100)
**Age, y**	
< 20	571 (39)
21-25	731 (50)
> 26	161 (11)
Total	1463 (100)
**Marital statu**s	
Single	1268 (86.7)
Married	178 (12.2)
Divorced	17 (1.2)
Total	1463 (100)

^a^ Data are presented in No. (%).

**Table 2.** Correlation Between Suicidal Ideation and Five Main Personality Factors

Personality Factors	Correlation Coefficient	Significance
**Neuroticism**	0.323	0.001
**Extraversion**	-0.306	0.001
**Openness to experience**	0.002	0.945
**Agreeableness**	-0.227	0.001
**Conscientiousness**	-0. 271	0.001

**Table 3.** Coefficient of Multiple Correlations Between Personality Factors and Suicidal Ideation

Stepwise Model	R	R^2^	Adjusted Coefficient of Determination	Standard Error Estimates
**Step 1**	0.323	0.10	0.10	1.92
**Step 2**	0.366	0.13	0.13	1.89
**Step 3**	0.371	0.14	0.14	1.88

**Table 4.** ANOVA, Significance of Two Models

	Sum of Squares	Degree of Freedom	Mean Squares	F	Significance
**Model 1**					0.001
Regression	566.258	1	566.258	152.968	
Residual	4849.363	1310	3. 702		
Total	5414.621	1311	-		
**Model 2**					0.001
Regression	724.026	2	362.013	101.005	
Residual	4691.595	1309	3.584		
Total	5415.621	1311	-		
**Model 3**					0.001
Regression	747.312	3	249.104	69.796	
Residual	4668.309	1308	3.569		
Total	5415.621	1311	-		

**Table 5.** Regression Coefficients

	Unstandardized Coefficients		Beta	t	Significance
	B	SE			
**Model 1**					
Constant value	-0.805	0.169	-	-4.769	0.001
Neuroticism	0.091	0.007	0.323	12.368	0.001
**Model 2**					
Constant value	1.497	0.385	-	3.890	0.001
Neuroticism	-0.065	0.008	0.229	7.782	0.001
Extraversion	0.060	0.009	-0.195	6.635	0.001
**Model 3**					
Constant value	1.906	0.416	-	4.582	0.001
Neuroticism	0.060	0.008	0.214	7.166	0.001
Extraversion	-0.046	0.011	-0.149	4.310	0.001
Conscientiousness	-0.023	0.009	0.086	2.554	0.011

The results given in Table 4, confirm the relationship between variables and the significance of three models are assured. Considering the coefficients of the three models, positive and significant relationships between all variables are assured. Also, the test statistic can be used to prove that neuroticism has a greater impact on prediction of suicide ideation than extraversion and conscientiousness.

 In Table 5, the common and standard regression analysis coefficients, standard deviation, statistic and the significance level of the model are presented. Based on these findings, the regression coefficients of three predictor variables of neuroticism, extraversion and conscientiousness may significantly predict suicidal ideation. Among these three features, neuroticism personality factor is positively associated with suicide ideation. This means that the more individuals have neuroticism personality factors, the more they are prone to commit suicide. Furthermore, extraversion and conscientiousness personality factors are negatively associated with suicidal ideation, and this means that the more individuals have these two traits, the less suicidal ideation may emerge.

**Figure 1.**
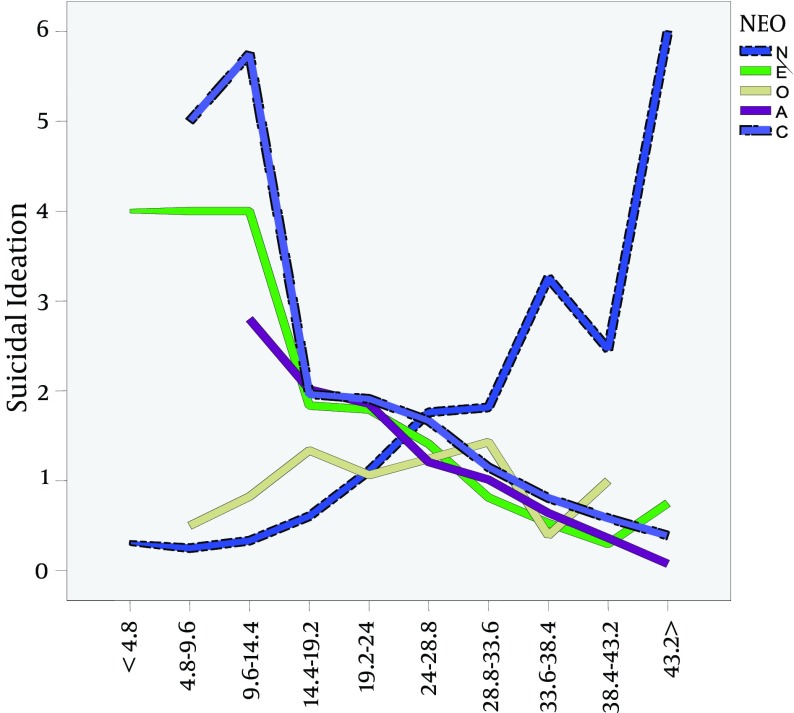
The Relationship Between Personality Traits and Suicidal Ideation A, agreeableness; C, conscientiousness E, extraversion; N, neuroticism; O, openness to experience.

According to the graph, suicidal ideation is associated with increased neurotics' features. The extraversion, conscientiousness, and agreeableness traits are in line with lower levels of suicidal ideation. Based on the coefficients, neuroticism, extraversion and conscientiousness personality factors, we are able to predict suicidal ideation. Formula to predict the suicidal thoughts are as follows;

*SI = 1.91 + *(*0.060 × NEON*)* -* (*0.046 × NEOE*)* - *(*0.023 × NEOC*).

## 5. Discussion

The main aim of this study was to investigate the role of personality factors in the prediction of suicidal ideation as a predisposing factor for suicides. Results obtained from the research sample revealed that neuroticism, extraversion and conscientiousness traits may predict suicidal ideation, but traits such as agreeableness and openness lack a predictive power; therefore, they were omitted from the model. 

These results are consistent with the studies of Andrea (14) and Rohtash (15). Personality factors increase the risk of suicide in two ways. First, some traits such as neuroticism may predispose individuals to psychiatric disorders such as depression and second; some traits such as impulsivity affect the way individuals react to the life events (11). Neuroticism has dimensions of depression, anger, hostility, impulsivity, and vulnerability. These factors, in addition to being directly involved in suicidal ideation, predispose the individual to suicide tendencies through creating despair. Despair as a risk factor for suicide, is positively correlated with neuroticism and negatively correlated with extraversion (14). In other words, Neuroticism is a predisposing factor for developing psychiatric disorders such as mood disorders, which may lead to suicide (19).

 Also, some studies have shown that neuroticism and changes in neurotransmitters involved in anxiety and depression are interrelated (20). But, extraversion is negatively related to suicidal behavior because extraversion is directly connected to vitality, joy and sociability (19). Extraversion is also negatively associated with despair and depression. Thus suicidal patients compared with those without suicidal behavior have lower extraversion scores. This finding is consistent with previous studies that reported the relationship of high neuroticism scores with a higher prevalence of suicidal ideation (21). 

Moreover, the results indicate the power of personality factors of extraversion and conscientiousness in the prediction of suicidal ideation (22). Conscientious personality factors have some dimensions, including competence, clarity, sufficiency, self-discipline, self-control and cautiousness in decision making. These features are negatively correlated with despair, depression, loneliness, lack of meaning and isolationism and because of their predisposing effects on suicidal behavior, logically there is a negative relationship between conscientiousness and suicidal tendencies (23). 

Also, conscientiousness components such as adequacy and competence have a positive relationship with self-sufficiency, joy and effective contrast. Likewise, studies have shown that, extraversion and conscientiousness are negatively associated with loneliness; individuals lose their presence in social networks just because of the lack of communication. Therefore, losing social support increases the risk of anxiety, depression and suicide (24). 

Based on the findings of this study, investigating soldiers' personality traits before deployment and use of psychological interventions, can reduce the risk of suicide in populations of young military sites.

The strong points of this study were collecting samples in six provinces of Iran, conducting research in military areas, using a powerful statistical modeling approach for analyzing the data, appropriate sample size, and homogeneity of the study population. Some weak points of this study were using self-report tools instead of psycho-physiological approach and probability of malingering in military camps.
